# Evaluation of retinal nerve fiber and ganglion cell layer abnormalities using optical coherence tomography in thyroid-associated orbitopathy

**DOI:** 10.5935/0004-2749.2024-0103

**Published:** 2025-06-24

**Authors:** Alisson Lima Andrade, Thiago Pereira Faria, Roberta Lilian Fernandes de Sousa Meneghim, Mitsuo Hashimoto, Edson Nacib Jorge, Silvana Artioli Schellini, Eliane Chaves Jorge

**Affiliations:** 1 Department of Surgical Specialties and Anaesthesiology, Ophthalmology Division, Faculdade de Medicina de Botucatu, Universidade Estadual Paulista “Júlio de Mesquita Filho”, Botucatu, SP, Brazil

**Keywords:** Graves’, ophthalmopathy, Optic nerve disorders, Re-tinal nerve fiber layer, Retinal ganglion cells, Optical coherence tomography

## Abstract

**Purpose:**

This study aimed to evaluate abnormalities in the retinal nerve fiber layer and ganglion cell layer in patients with thyroid-associated orbitopathy using optical coherence tomography and to examine their relationship with disease severity.

**Methods:**

A cross-sectional study was conducted involving 74 participants, comprising 45 individuals with thyroid-associated orbitopathy and 29 healthy controls. All subjects underwent a comprehensive ophthalmological examination and optical coherence tomography using the Cirrus HD-OCT. The clinical activity score and the European Group on Graves’ Orbitopathy severity were also evaluated.

**Results:**

In the thyroid-associated orbitopathy group, the mean peripapillary retinal nerve fiber layer thickness was significantly reduced in the temporal quadrant (p<0.05). No significant differences were found in ganglion cell layer thickness across all sectors when compared with the control group. Besides, a significant correlation was observed between orbitopathy severity and decreased mean peripapillary retinal nerve fiber layer thickness (p<0.001).

**Conclusion:**

Optical coherence tomography may serve as a useful tool for identifying changes in the retinal nerve fiber layer and ganglion cell layer in patients with thyroid-associated orbitopathy, including in the inactive phase and prior to the clinical manifestation of dysthyroid optic neuropathy. It may be a helpful adjunct in monitoring disease progression.

## INTRODUCTION

Thyroid-associated orbitopathy (TAO) is an autoimmune condition and the most common extra--thyroidal manifestation of Graves’ disease (GD)^(1)^. It is characterized by an initial active or inflammatory phase that primarily affects the orbital soft tissues, including fat, extraocular muscles, and the optic nerve, followed by a prolonged inactive phase^(2^,^3)^.

TAO is usually bilateral, often asymmetric, and generally mild. However, in 5%-8% of cases, it can progress to a severe form known as dysthyroid optic neuropathy (DON). In approximately 90% of these cases, DON results from optic nerve compression due to enlarged extraocular muscles at the orbital apex, while about 10% are attributed to optic nerve stretching^(4^,^5)^. This serious complication of TAO necessitates timely evaluation and management to avoid permanent vision loss^(6)^.

Spectral-domain optical coherence tomography (SD-OCT) is a noninvasive diagnostic method that can be used to evaluate TAO activity and detect early signs of DON^(7^,^8)^. Previous studies suggest that measuring the thickness of the retinal nerve fiber layer (RNFL) and ganglion cell layer (GCL) may help identify structural impairment, even during the inactive phase of TAO^(9)^. OCT findings in individuals with TAO vary, with some studies reporting thickening of the RNFL and choroid, while others have observed RNFL thinning in these patients^(10^-^14)^. Based on this, the current study aims to compare RNFL and GCL thickness between TAO patients and healthy controls and to examine their correlation with the severity of orbitopathy.

## METHODS

A prospective, cross-sectional, controlled study was carried out at the University Hospital of Botucatu Medical School- UNESP, Brazil, between October 2020 and July 2021. The study protocol was approved by the Research Ethics Committee (approval no. 3553947), and all participants provided written informed consent in accordance with the Declaration of Helsinki.

The study involved two groups: a TAO group and a control group. Inclusion criteria for the TAO group were individuals over 18 years of age with a clinical diagnosis of TAO, regardless of gender, ethnicity, or disease stage. For the control group, inclusion criteria were individuals over 18 years old, regardless of gender or ethnicity, with best-corrected visual acuity (BCVA) >20/30 and intraocular pressure (IOP) <21 mmHg, and without a diagnosis of GD or any intraocular pathology.

Exclusion criteria included any conditions that could affect OCT measurements, such as amblyopia, glaucoma, optic nerve or retina disorders, media opacities, and strabismus. The Clinical Activity Score (CAS) was used to assess disease activity based on signs including conjunctival and eyelid redness and swelling, as well as retro-orbital pain; a CAS of ≥3 out of 7 was considered indicative of active TAO. Clinical features were assessed using the criteria established by the European Group on Graves’ Orbitopathy (EUGOGO), which include quality of life impairment, proptosis measurement, lid retraction, diplopia, signs of optic neuropathy, or corneal involvement^(15)^, and the disease was classified as mild, moderate-to-severe, or very severe (sight-threatening)^(15)^.

DON was suspected when a combination of findings was present, including reduced visual acuity, relative afferent pupillary defect, abnormal color vision, visual field defects, optic disc swelling or atrophy, or evidence of orbital apex crowding.

Data collection included demographic information, clinical evaluation, and a comprehensive ophthalmological examination. This involved BCVA (converted to logMAR for statistical analysis), biomicroscopy, exophthalmometry, color vision testing using the Ishihara test, IOP measurement, and indirect fundoscopy. Extrao-cular muscle thickness was measured using computed tomography (CT) OPTIMA 64 (GE Healthcare, Arlington Heights, USA), with contiguous axial and direct coronal slices of 0.5-1 mm through the orbit. SD-OCT was performed using the Cirrus HD-OCT (Carl Zeiss Meditec Inc., Germany) to evaluate peripapillary RNFL thickness. This was done through three consecutive 360^o^ scans centered on the optic disc with a diameter of 3.4 mm, each composed of 256 A-scans acquired in a single session. Mean RNFL thickness was analyzed in four quadrants: superior, inferior, nasal, and temporal (each covering 90^o^). GCL thickness was measured using the Macular Cube 512 × 128 protocol, focusing on the area between the GCL and the inner plexiform layer (IPL). Inclusion criteria for scans required a signal strength of ≥6, absence of motion artifacts or segmentation errors, and proper centration on the fovea.

All data were entered into an Excel spreadsheet and analyzed using the SAS^®^ software for Windows (version 9.4, SAS Inc.). The Student’s t-test was used to compare the mean RNFL thickness between the TAO group and the control group. To assess the association between RNFL thickness and ethnicity, only the measurement from the right eye was considered. Variables that did not follow a normal distribution were analyzed using a generalized linear model with a gamma distribution. Discrete variables were evaluated using a Poisson regression model. The chi-squared test was used to examine associations between categorical variables and groups. Spearman’s correlation was used to assess the relationship between RNFL thickness and visual parameters, including dyschromatopsia, logMAR visual acuity, and fundoscopic findings. The correlation between TAO severity and OCT measurements was analyzed and adjusted for potential confounders using the ANOVA test. A p-value <0.05 was considered statistically significant.

## RESULTS

The study included 89 eyes from 45 TAO patients and 58 eyes from 29 control participants. The average age was 49.3 ± 14 years for the TAO group and 49.7 ± 14.9 years for the control group. Among the TAO patients, 35 (77.8%) were female, while in the control group, 24 (82.8%) were female. There were no significant differences between the groups regarding age (p=0.90) and gender (p=0.63). Most participants identified as white (66,2%). One eye from the TAO group was excluded due to media opacity.

Systemic arterial hypertension was the most common comorbidity (37.8% in TAO vs. 31% in the control group), followed by diabetes (20% in TAO vs. 17% in the control group) and smoking (13.3% in TAO vs. 3.4% in the control group), with no significant difference between the groups.

Of the 45 TAO patients, 42 (93.3%) were in the inactive phase, with a mean CAS of 1.1 and an average disease duration of 6.6 years. Thirty patients had mild TAO, 12 had moderate-to-severe TAO, and 3 had sight-threatening disease. BCVA and mean IOP did not differ significantly between the TAO and control groups (p>0.05) (Table 1).

**Table 1:** Demographic information and clinical characteristics of TAO patients and healthy controls

	TAO group (n=45)	Healthy group (n=29)	p-value
Mean age (year±SD)	49.3 ± 14.0	49.7 ± 14.9	0.90
Gender (female)	35 (77.8%)	24 (82.8%)	0.63
BCVA (logMAR)	0.04 ± 0.1	0.02 ± 0.06	0.15
Exophthalmometry (mm±SD)	20.1 ± 2.86 mm	14.3 ± 1.8 6 mm	<0.05^*^
IOP (mmHg ± SD)	13.5 ± 2.32 mmHg	13.0 ± 2.03 mmHg	>0.05
Dyschromatopsia	4 (8.9%)	0 (0%)	>0.05

BCVA= best-corrected visual acuity; TAO= thyroid-associated orbitopathy; IOP= intraocular pressure; SD= standard deviation;

* p-value <0.05.

The Ishihara test showed abnormalities in only four patients (8.9%) in the TAO group, and no changes were noted in the fundoscopy.

The mean exophthalmometry measurement was significantly higher in the TAO group (20.1 ± 2.86 mm) compared to the control group (14.3 ± 1.86 mm; p<0.05) (Table 1). CT imaging of extraocular muscle thickness was performed in 31 TAO patients, with 3 of them having undergone orbital decompression.

The mean RNFL thickness was lower in the TAO group (89.1 ± 11.8 µm) compared to the control group (92.4 ± 8.3 µm), though this difference was not statistically significant (p=0.06). However, RNFL thickness was significantly reduced in the temporal quadrant of the TAO group compared to the control group (TAO, 57.8 ± 10.5 µm vs. control, 63.0 ± 11.3 µm; p<0.05) (Table 2).

**Table 2:** Retinal nerve fiber layer measurements (overall mean and sectoral) in TAO patients and healthy controls

	TAO group (n=45)	Healthy group (n=29)	p-value
RNFL total	89.1 ± 11.8	92.4 ± 8.3	0.06
RNFL superior	112.4 ± 19.3	115.3 ± 14.9	0.32
RNFL inferior	119.8 ± 19.7	122.3 ± 13.9	0.40
RNFL nasal	67.9 ± 13.0	67.4 ± 11.0	0.81
RNFL temporal	57.8 ± 10.5	63.0 ± 11.3	0.005^*^

RNFL= retinal nerve fiber layer; TAO= thyroid-associated orbitopathy;

* p-value <0.05.

A strong correlation was found between the severity of orbitopathy and the reduction in mean RNFL thickness (p<0.0001), except in the nasal sector (p=0.06), after adjusting for sex and age (Table 3). Additionally, no significant differences were observed in mean GCL thickness between the TAO and control groups (Table 4). However, a correlation was noted between TAO severity and reduced mean GCL thickness (Table 5), particularly in the temporal sector (p<0.0001).

**Table 3:** Comparison between RNFL measurements and the EUGOGO severity score

	Mild TAO	Moderate-to-severe TAO	Sight-threatening TAO	p-value
RNFL total	89.5 ± 7.4	94.2 ± 13.9	62.8 ± 12.8	<0.0001^*^
RNFL superior	112.8 ± 13.6	118.9 ± 23.7	76.6 ± 19.1	<0.0001^*^
RNFL inferior	118.6 ± 13.7	130.2 ± 21.5	83.8 ± 28.2	<0.0001^*^
RNFL nasal	69.1 ± 11.8	67.3 ± 14.3	55.0 ± 16.7	0.06
RNFL temporal	57.8 ± 10.4	60.3 ± 8.9	45.0 ± 12.5	0.01^*^

TAO= thyroid-associated orbitopathy; RNFL= retinal nerve fiber layer;

* p-value <0.05.

**Table 4:** Ganglion cell layer measurements (overall mean and sectoral) in TAO patients and healthy controls

	TAO group (n=45)	Healthy group (n=29)	p-value
GCL total	79.1 ± 10.9	81.0 ± 6.6	0.35
GCL TS	80.4 ± 9.9	81.1 ± 8.6	0.74
GCL S	80.1 ± 12.8	82.3 ± 6.6	0.35
GCL NS	79.1 ± 13.0	81.4 ± 7.5	0.36
GCL NI	78.4 ± 13.3	81.6 ± 6.3	0.18
GCL I	77.3 ± 13.2	79.7 ± 8.2	0.35
GCL TI	79.3 ± 13.0	81.0 ± 9.4	0.49

TAO= thyroid-associated orbitopathy; GCL= ganglion cell layer; TS= temporal and superior; S= superior; NS= nasal and superior; NI= nasal and inferior; I= inferior; TI= temporal and inferior;

* p-value <0.05.

**Table 5:** Comparison between GCL measurements and the EUGOGO severity score

	Mild TAO	Moderate-to-severe TAO	Sight-threatening TAO	p-value
GCL total	80.9 ± 9.8	80.7 ± 6.9	57.3 ± 10.1	<0.0004^*^
GCL TS	82.4 ± 8.0	80.9 ± 6.7	57.7 ± 11.5	<0.0001^*^
GCL S	81.9 ± 12.2	82.1 ± 7.3	57.0 ± 8.9	<0.002^*^
GCL NS	80.6 ± 13.3	80.9 ± 6.9	61.0 ± 6.3	0.028^*^
GCL NI	80.2 ± 13.4	79.9 ± 14.7	58.0 ± 10.8	0.013^*^
GCL I	79.2 ± 12.6	78.9 ± 7.4	55.0 ± 14.7	0.005^*^
GCL TI	81.2 ± 12.5	80.7 ± 7.5	57.3 ± 12.2	0.005^*^

TAO= thyroid-associated orbitopathy; GCL= ganglion cell layer; TS= temporal and superior; S= superior; NS= nasal and superior; NI= nasal and inferior; I= inferior; TI= temporal and inferior;

* p-value <0.05.

Further analysis showed that ethnicity was not asso-ciated with RNFL thickness (p=0.33), while poorer visual acuity was weakly correlated with decreased RNFL thickness (r=-0.317; p<0.001). Dyschromatopsia was also linked to RNFL thinning (r=-0.323; p<0.0001).

## DISCUSSION

This study highlights abnormalities in RNFL and GCL measurements using SD-OCT in TAO patients, showing a positive correlation with disease severity.

Our sample shares similar characteristics with previous studies, such as a higher proportion of women^(10^,^16)^ and a majority of inactive cases without visual impairment^(17)^. Although most patients were in the inactive phase and did not show signs of neuropathy, OCT evalua-tion revealed a significant reduction in RNFL thickness, particularly in the temporal quadrant.

Previous studies have also reported a sectoral reduction in peripapillary RNFL in TAO patients, with many being in the inactive phase^(8^,^9^,^13^,^14)^. Decreased temporal RNFL thickness has been observed in very severe TAO, as well as in acute or chronic optic neuropathy^(18)^, suggesting that RNFL changes can occur even during the quiescent phase of TAO.

This study’s analysis of total peripapillary thickness (including all four quadrants) did not show a statistical difference between the TAO and control groups. However, a lower thickness was observed in the TAO group.

The predominance of inactive TAO patients (93.3%) may have contributed to the small difference between the mean and sectoral RNFL thicknesses. This finding aligns with previous studies that analyzed full-thickness RNFL in TAO patients and healthy controls^(19)^.

Regarding the relationship between TAO severity and RNFL thickness, previous studies have reported varying results^(10^,^13^,^14)^. Our study found a strong correlation between orbitopathy severity and reduced mean RNFL thickness, consistent with the findings of Kurt et al., Mugdha et al., and Luo et al.^(13^,^14^,^17)^. In contrast, Blum-Meirovitch et al. observed RNFL thickening in the nasal, superior, and inferior sectors, while the temporal sector remained normal^(10)^. These differences may be attributed to the higher proportion of active and severe cases in the Blum-Meirovitch study and others, where RNFL thickening may be associated with the extent of inflammation and optic nerve and orbital swelling in patients with active or severe TAO^(9^,^10^,^20)^.

Interestingly, OCT angiography (OCT-A) studies have shown an increase in thickness and a decrease in peripapillary vascular density in eyes with active TAO, a pattern not observed in eyes without activity^(21)^. However, individuals with moderate-to-severe TAO demonstrated a reduction in mean peripapillary RNFL thickness in the nasal and temporal sectors. Conversely, those with sight-threatening TAO showed thickened RNFL in all quadrants^(17)^.

These findings suggest that TAO may exhibit a disconnect between the structural and functional aspects of the optic nerve^(8)^, with structural damage likely occurring before functional impairment. In the current study, patients with more severe TAO did not show visible fundoscopic changes in the optic nerve but exhibited signs of chronic DON process, leading to subclinical RNFL atrophy. This may explain the weak correlation between dyschromatopsia and RNFL thinning.

In line with previous studies, the GCL/IPL thickness was thinner in patients with sight-threatening TAO compared to those with mild or moderate-to-severe orbitopathy in our study^(9^,^22)^.

The limitations of our study include the relatively small sample size and the low number of patients with severe TAO. Additionally, the absence of data on visual field and refractive errors limited our analysis.

Our study emphasizes RNFL changes in TAO patients, comparing orbitopathy severity and OCT measurements in our racially diverse population. However, no difference in RNFL were observed in relation to race due to the small percentage of self-identified Black and Asian participants.

Our findings suggest that OCT may be a useful tool for detecting changes in peripapillary RNFL and GCL in inactive TAO patients, even in the absence of clinical sign of DON, as OCT can reveal a reduction in RNFL^(13^,^14^,^17^,^23)^.

The key finding was the detection of RNFL abnormalities, even in the most inactive and mild cases of TAO. However, further research and prospective studies are needed to confirm these results and monitor the long-term effects of TAO on the optic nerve in our population.
